# The influence of the windlass mechanism on kinematic and kinetic foot joint coupling

**DOI:** 10.1186/s13047-022-00520-z

**Published:** 2022-02-16

**Authors:** Lauren R. Williams, Sarah T. Ridge, A. Wayne Johnson, Elisa S. Arch, Dustin A. Bruening

**Affiliations:** 1 Brigham Young University, Provo, UT 84602 USA; 2 University of Delaware, Newark, DE 19716 USA

**Keywords:** Medial longitudinal arch, Metatarsophalangeal joint, Multisegment foot, Foot energetics, Heel raise

## Abstract

**Background:**

Previous research shows kinematic and kinetic coupling between the metatarsophalangeal (MTP) and midtarsal joints during gait. Studying the effects of MTP position as well as foot structure on this coupling may help determine to what extent foot coupling during dynamic and active movement is due to the windlass mechanism. This study’s purpose was to investigate the kinematic and kinetic foot coupling during controlled passive, active, and dynamic movements.

**Methods:**

After arch height and flexibility were measured, participants performed four conditions: Seated Passive MTP Extension, Seated Active MTP Extension, Standing Passive MTP Extension, and Standing Active MTP Extension. Next, participants performed three heel raise conditions that manipulated the starting position of the MTP joint: Neutral, Toe Extension, and Toe Flexion. A multisegment foot model was created in Visual 3D and used to calculate ankle, midtarsal, and MTP joint kinematics and kinetics.

**Results:**

Kinematic coupling (ratio of midtarsal to MTP angular displacement) was approximately six times greater in Neutral heel raises compared to Seated Passive MTP Extension, suggesting that the windlass only plays a small kinematic role in dynamic tasks. As the starting position of the MTP joint became increasingly extended during heel raises, the amount of negative work at the MTP joint and positive work at the midtarsal joint increased proportionally, while distal-to-hindfoot work remained unchanged. Correlations suggest that there is not a strong relationship between static arch height/flexibility and kinematic foot coupling.

**Conclusions:**

Our results show that there is kinematic and kinetic coupling within the distal foot, but this coupling is attributed only in small measure to the windlass mechanism. Additional sources of coupling include foot muscles and elastic energy storage and return within ligaments and tendons. Furthermore, our results suggest that the plantar aponeurosis does not function as a rigid cable but likely has extensibility that affects the effectiveness of the windlass mechanism. Arch structure did not affect foot coupling, suggesting that static arch height or arch flexibility alone may not be adequate predictors of dynamic foot function.

**Supplementary Information:**

The online version contains supplementary material available at 10.1186/s13047-022-00520-z.

## Background

The energetic role of the foot in gait is critical to our understanding of foot function and in applications such as footwear, assistive devices (e.g. orthotics) and prosthetics. However, the foot’s complex structure has traditionally made it difficult to model, resulting in overly simplified perspectives regarding its role in locomotion energetics. Early theoretical models of the foot’s role in gait, such as the midtarsal locking theory [1] and the twisted footplate model [2], highlight the idea that the foot stiffens to act as a rigid lever for propulsion in late stance. This perspective is still frequently disseminated (e.g. [3, 4]) despite many multisegment foot studies challenging this viewpoint. Instead, these studies have shown that substantial medial longitudinal arch (MLA) rise occurs [5–9] and the midtarsal joint generates considerable power during push-off [5, 10–13]. As power is generated at the midtarsal joint, power is simultaneously absorbed at the 1st metatarsophalangeal (MTP) joint [11–14] suggesting both kinematic (e.g. [15, 16]) and kinetic coupling between these two joints [12, 13, 17]. In addition, changes in MTP kinematics and kinetics due to task manipulation (e.g. varying footstrike pattern [5] or walking speed [18] show proportional changes at the midtarsal joint, further reinforcing this coupling.

Intersegment coordination, or coupling, between the MTP and midtarsal joints can be facilitated by the numerous muscles, ligaments, and other connective tissues that span both joints [19, 20]. One of the main passive structures linking these joints is the plantar aponeurosis (PA), a critical component of the windlass mechanism [21]. This mechanism has been demonstrated passively [22, 23], where toe extension induces tension in the PA which draws the calcaneus and head of the first metatarsal together, effectively causing the midtarsal joint to plantarflex (i.e., the MLA rises). Dynamically, when the MTP joint extends during late stance, this induced tension could transfer energy from the MTP joint to the midtarsal joint, facilitating positive midtarsal power generation during push-off and increasing gait efficiency [12, 13, 17]. However, a number of studies have highlighted the importance of muscular actions as a source of midtarsal power [24–26], and the role of the windlass mechanism in locomotion is currently not fully known. A systematic manipulation of MTP mechanics may help determine to what extent this distal foot coupling is due to the windlass mechanism versus active muscle contractions or springlike tissues.

Foot structure may be correlated with how well the windlass mechanism functions, and therefore also possibly correlated with foot energetics. For instance, Lucas et al. observed qualitatively that individuals with an impaired windlass mechanism (i.e., delayed or absent midtarsal rise with passive MTP extension) have greater arch mobility and lower arches compared to individuals with an intact windlass mechanism [27], perhaps due to a combination of bony structure and joint position, ligamentous laxity, and muscle strength/control. Wilken et al. showed that arch height had a modest relationship with proximal foot joint coupling measurements during terminal stance [28], suggesting an influence from the windlass mechanism. Studying the effect of differences in arch flexibility on kinematic coupling may provide additional insight to help distinguish between passive and dynamic coupling sources.

The overall purpose of this study was to deconstruct the kinematic and kinetic coupling between the MTP and midtarsal joints through a systematic investigation influencing the windlass mechanism. First, we aimed to determine limits on passive kinematic coupling from the windlass mechanism, by manipulating MTP motion while seated, standing, and during heel raises. We hypothesized that the ratio of midtarsal to MTP motion would increase from seated and standing to dynamic tasks [23]. Secondly, we aimed to confirm kinetic coupling between the MTP and midtarsal joints during dynamic heel raises. We hypothesized that as MTP motion was manipulated changes in MTP joint negative work would be proportional to opposing changes in midtarsal positive work [5], while distal-to-hindfoot work (which covers the net contributions of all foot joints distal to the hindfoot [12]) would remain constant across all conditions. The final aim was to explore the relationship between foot structure measures and foot coupling during the same passive and dynamic movements. We hypothesized that low arch height and high arch flexibility would be correlated with a less active windlass mechanism [27] (i.e., less midtarsal motion per degree of MTP extension). If the windlass mechanism does influence dynamic coupling between the MTP and midtarsal joints, this knowledge could enhance our understanding of human gait energetics, influence clinical treatment of gait deficiencies, and inform the design of assistive foot devices.

## Methods

### Participants

Twenty-eight individuals (11 female, 17 male; age: 24.3 ± 4.6; height: 1.75 ± 0.07 m; body mass: 74.6 ± 12.8 kg) volunteered to participate in this randomized cross-over study. Participants were excluded if they had a history of musculoskeletal or neurological disease, had undergone any surgery in the lower extremities, or had a serious lower extremity injury that resulted in an inability to resume all previous physical activities. Before any data collection, participants were asked to thoroughly read and sign an IRB-approved informed consent form.

### Procedures

First, investigators used the Arch Height Index Measurement System (AHIMS; JAK Tool & Model, NJ, USA) to determine the height and flexibility of the participant’s arch. Only the left foot was measured, as there is no difference between left and right sides for arch height index measurements [29, 30], and lab set-up made the left foot the preferred side on which to perform all further testing. Arch flexibility was calculated using the following equation, where ‘AH’ is the height of the foot’s dorsum from the floor at half of the total foot length, and ‘BW’ is body weight [30]:

$$ Arch\ Flexibility\ \left(\frac{cm}{kg}\right)=\frac{AH_{sitting}-{AH}_{standing}}{0.4\ast BW}\ast 100 $$

In order to get arch flexibility into the same units reported by Zifchock et al. (mm/kN) [30], the value calculated from this equation was multiplied by 10,000 and divided by 9.8. A low arch flexibility value indicates a stiff arch, and a high arch flexibility value indicates a flexible arch. For this study, we targeted participants with varying foot structures so that we had a range of arch heights and flexibilities. Participant’s height and weight were also measured.

After all preliminary data was obtained, the left foot was outfitted with a multisegment foot model. The marker set used for this study closely resembled the multisegment foot model developed by Bruening et al. [11], but with a few modifications (Marker placement and model description can be found in Additional file 1). Following marker placement, participants performed four order-randomized tasks where isolated MTP extension was achieved either passively or actively: 1) Seated Passive MTP Extension, 2) Seated Active MTP Extension, 3) Standing Passive MTP Extension, and 4) Standing Active MTP Extension. During the passive trials, an investigator passively extended the participant’s MTP joint to its end range of motion 10 consecutive times for two sets to a metronome of 40 beats per minute. Standing Passive MTP Extension was achieved by having the participants stand on a block two feet off the ground and placing their feet so that their toes were off the edge. This set-up allowed the investigator to push the MTP joint into extension without blocking the view of the motion capture cameras. During the active trials, participants were instructed to extend their 1st MTP joint as far as possible to the metronome. Set up during Standing Active MTP Extension was identical to Standing Passive MTP Extension. These isolated tasks allowed us to investigate with motion capture the kinematic coupling that occurs between MTP and midtarsal joints when the windlass mechanism is passively and actively engaged.

**Fig. 1:**
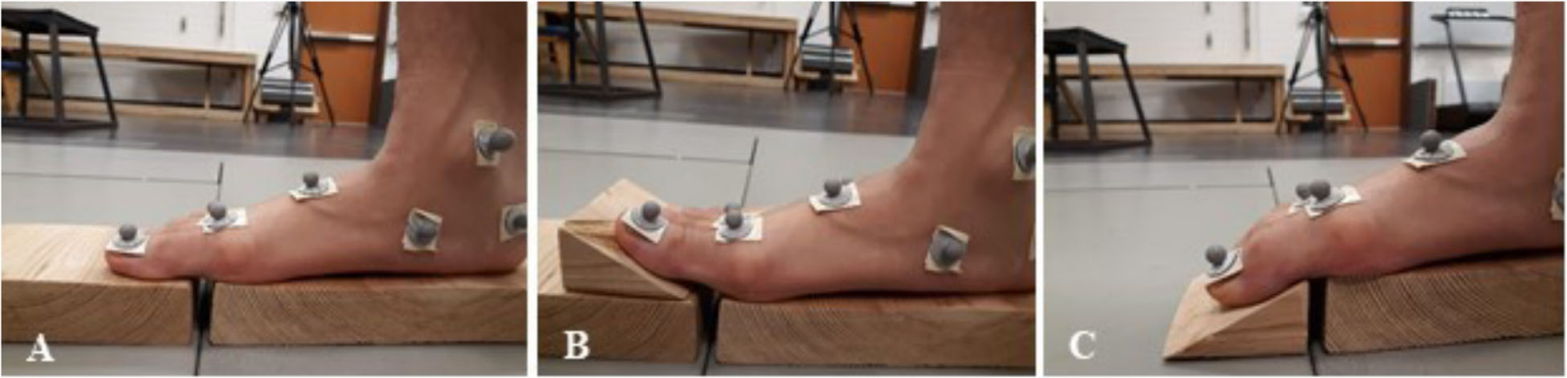
Heel Raise Conditions **A**. Neutral heel raise. **B**. ToeExt heel raise. **C**. ToeFlex heel raise

Next, participants performed three double-leg heel raise conditions that manipulated the starting position of the MTP joint during dynamic movement: 1) Neutral: normal heel raises (control), 2) ToeExt: heel raises with the toes placed on an inclined surface of 30 degrees to put the MTP joint into extension, and 3) ToeFlex: heel raises with the toes placed on a declined surface of 30 degrees to put the MTP joint into flexion. To achieve the inclined and declined surfaces, blocks were placed on two adjacent in-ground force plates (Fig. 1). The toes were placed on the angled surface mounted to one force plate, while the rest of the foot was placed on a flat surface on the other plate. The foot segments were placed on different force plates to partition the ground reaction forces under each segment [14]. Force plates were zeroed after the blocks were mounted and between each condition. All heel raise conditions had two sets of 10 consecutive trials and were performed to a metronome of 40 beats per minute to control ankle angular velocity. A tripod was placed in front of participants during heel raises, which they could lightly touch with their fingertips to help them maintain balance during the different conditions if needed. To help with achieving the same height across all three conditions, participants wore a headband with a marker secured to the top and tried to match the height of this marker with a target marker visible from an orthogonal front view on a screen in front of them. The position of this target marker did not change between conditions. Heel raises were used as a dynamic task because they provide a controlled environment to systematically manipulate the MTP joint. Motion at the midfoot and ankle as well as ankle power during heel raises and push-off during walking are significantly correlated [31], suggesting that heel raises may serve as an adequate surrogate for the push off phase of gait. These controlled heel raises allowed us to evaluate associated changes in foot kinematics and energetics during a dynamic task where the windlass mechanism is dynamically engaged.

### Data analysis

Kinematic data were collected at 200 Hz and low-pass filtered (Butterworth) at 6 Hz, while kinetic data were captured at 1000 Hz and low-pass filtered at 50 Hz. Motion capture trajectories were exported from Qualisys Track Manager software (Qualisys, Goteborg, Sweden) and imported into Visual 3D software (C-Motion, Inc., Germantown, MD, USA). After all data was imported into Visual 3D, a multisegment foot model was created [11], and ankle, midtarsal, and MTP joint angles, moments and powers (including distal-to-hindfoot power [32]) were calculated. Angles were calculated using a typical Euler/Cardan rotation sequence (1-flex/ext., 2-ab/ad, 3-int/ext. rotation). Work was calculated as the integration of the power curve during the upward phase of the heel raise. To account for small changes in the height achieved across the heel raise conditions, work was calculated from the start of the heel raise to the lowest height achieved during any of the three heel raise conditions. All kinetic variables were scaled by body mass.

To measure the amount of kinematic coupling that occurs between the MTP and midtarsal joints, the slope of the line (linear regression fit) created by plotting the mean angle curve (across cycles) from neutral to peak MTP extension for the midtarsal joint versus the MTP joint was calculated. During pilot testing, we confirmed that this relationship was linear and recent work also shows a linear relationship between midtarsal and MTP joint motion [23]. This linear slope (known from now on as ‘distal foot coupling ratio’ or DFCR; (ΔMidtarsal Angle) / (ΔMTP Angle)) was then used as a metric to explain the amount of kinematic coupling between these two joints during both the dynamic (i.e. heel raises) MTP extension conditions and the isolated (i.e. passive/active/seated/standing) MTP extension conditions.

### Statistical analysis

To determine if the amount of kinematic coupling changed across all conditions, a repeated measures analysis of variance (ANOVA) was done to compare the DFCR metric from each condition. A series of repeated measures ANOVAs were done for MTP negative work, midtarsal positive work, distal-to-hindfoot work, and ankle positive work across the three heel raise conditions. For each ANOVA, Mauchly’s test for sphericity was tested and corrected for if necessary. A Holm post hoc test was applied if the main effect showed significance (α = 0.05). A series of correlations (α = 0.05) were conducted for each condition to assess the relationship between arch flexibility and standing arch height and DFCR.

## Results

### Kinematic coupling: distal foot coupling ratio across all conditions

**Fig. 2:**
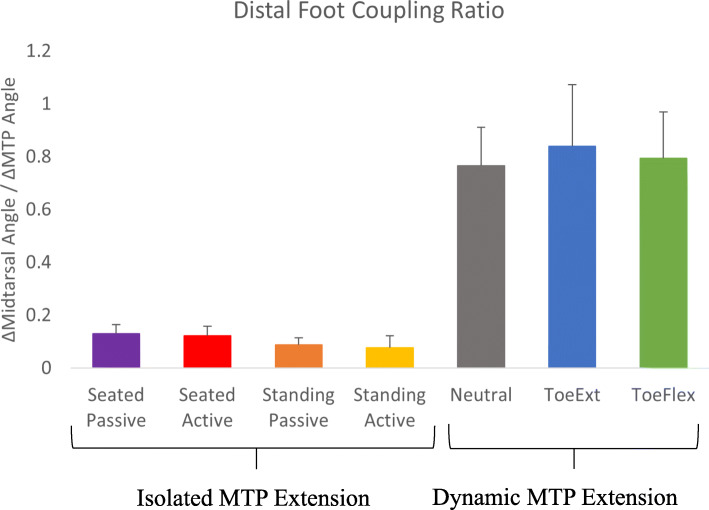
Distal Foot Coupling Ratio for All Conditions. Distal foot coupling ratio (∆Midtarsal Angle / ∆MTP Angle). Bars represent means with standard deviation error bars across all conditions. Dynamic MTP Extension conditions had greater coupling ratios than all the Isolated MTP Extension conditions (*p* < 0.001). Within the Isolated MTP Extension conditions, both seated conditions had significantly greater DFCR than both standing conditions (*p* < 0.05)

The relationship between midtarsal and MTP joint motion was linear for all conditions, allowing the DFCR metric to be calculated and used for analysis. The repeated measures ANOVA analyzing DFCR across all conditions had a significant main effect (*p* < 0.001). The post hoc analysis revealed that all the dynamic MTP extension conditions (Neutral: 0.765 ± 0.15, ToeExt: 0.838 ± 0.233, ToeFlex: 0.794 ± 0.126) had a larger DFCR than the isolated MTP extension conditions (p < 0.001; Fig. 2). There was no statistical difference in DFCR between heel raise conditions. Among isolated MTP extension conditions, both standing conditions (Standing Passive: 0.087 ± 0.03, Standing Active: 0.077 ± 0.04) had less coupling compared to both seated conditions (Seated Passive: 0.130 ± 0.03, Seated Active: 0.122 ± 0.04; Fig. 2; *p* < 0.01), but there was no difference between the active and passive MTP extension within seated or standing conditions.

### Kinetic coupling: joint work during heel raises

**Fig. 3:**
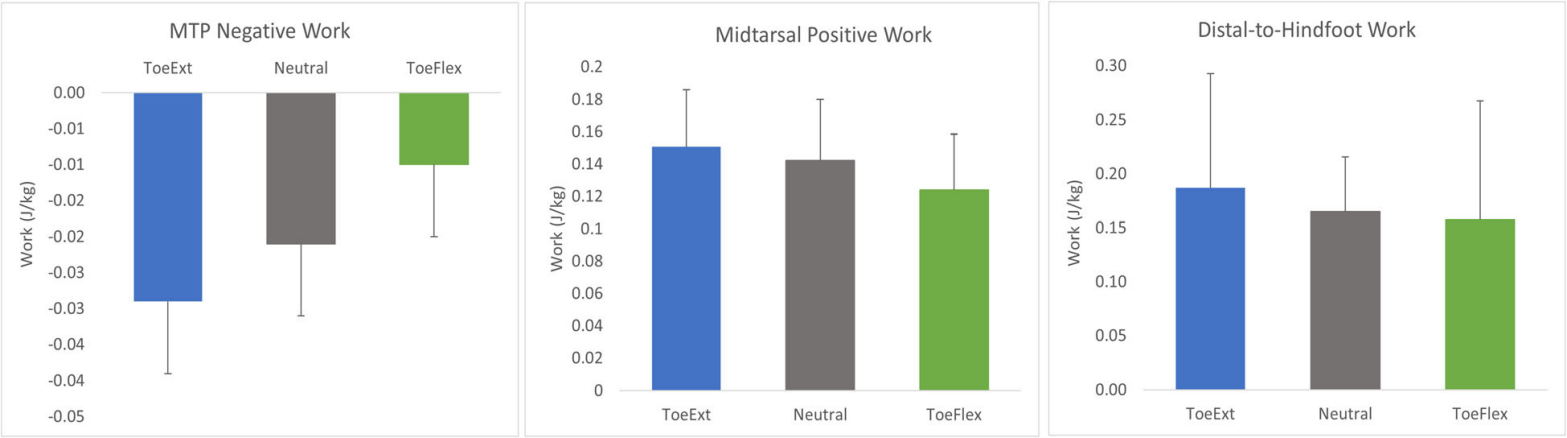
MTP, Midtarsal, and Distal-to-Hindfoot Work During Heel Raise Conditions. ToeExt, Neutral, and ToeFlex heel raise conditions. Bars represent means with standard deviation error bars. All conditions were significantly different (*p* < 0.01) for MTP and Midtarsal, while there was no significant difference between conditions for Distal-to-Hindfoot work

As the starting position of the MTP joint became increasingly extended, the amount of negative work done at the MTP joint increased (ToeExt: − 0.029 ± 0.01, Neutral: − 0.021 ± 0.009, ToeFlex: − 0.01 ± 0.008; *p* < 0.001; Fig. 3). Similarly, midtarsal positive work was greatest for ToeExt and smallest for ToeFlex (ToeExt: 0.151 ± 0.035, Neutral: 0.142 ± 0.038, ToeFlex: 0.124 ± 0.035); *p* < 0.01; Fig. 3). Distal-to-hindfoot work was not significantly different between Neutral, ToeExt, and ToeFlex (Fig. 3). Additionally, ankle positive work was not significantly different across all three conditions, indicating that the ankle did not compensate for changes at the MTP joint.

### Correlation: arch flexibility and arch height versus distal foot coupling ratio

The average standing arch height index was 0.33 ± 0.02, ranging from 0.29 to 0.38. The average arch flexibility was 13.99 ± 5.9 mm/kN, ranging from 3.52 to 27.19 mm/kN.

Of the isolated MTP extension conditions, arch flexibility was moderately and negatively correlated with DFCR during Seated Active MTP Extension (r = − 0.42, *p* = 0.03), indicating that as arch flexibility increases, the amount of midtarsal plantarflexion per degree of MTP extension decreases. Standing arch height index was weakly and positively correlated with DFCR during Standing Active MTP Extension (r = 0.39, *p* = 0.04). However, all other isolated conditions were not significantly correlated with arch flexibility or standing arch height.

Of the dynamic conditions, arch flexibility was moderately and positively correlated with DFCR during Neutral (r = 0.402, *p* = 0.03) and only weakly correlated with DFCR during ToeFlex (r = 0.369, *p* = 0.05). Arch height was not significantly correlated with DFCR during heel raises.

## Discussion

The overall purpose of this study was to investigate coupling within the distal foot during tasks of varying complexity and MTP positioning, with the goal of garnering greater understanding of the passive windlass mechanism’s role during dynamic movement. In support of our first hypothesis, the ratio of midtarsal to MTP motion increased from isolated MTP extension to dynamic MTP extension. Our kinematic results help determine limits on passive kinematic coupling, and are in line with a recent similar study [23], but with some important differences (detailed below). Regarding kinetic coupling, our hypothesis that changes in MTP joint negative work would be proportional to changes in midtarsal positive work was also met, and the differences in kinematic and kinetic coupling provide additional insights into distal foot energy sources. And lastly, our investigation of foot structural differences suggests that there is not a strong relationship between foot structure and kinematic foot coupling.

### Kinematic coupling and task complexity

Any midtarsal motion captured during Seated Passive MTP Extension is likely due almost entirely to the MTP extension itself (i.e., the windlass mechanism). When sitting in a non-weight bearing position, the plantar intrinsic foot muscles are inactive [33]. While it is possible that even passive extension of the toes could evoke a small muscular response, care was taken to ensure investigators felt no active assistance or resistance when pushing the MTP joint into extension. If motion at the midtarsal joint was entirely due to the windlass mechanism during Seated Passive MTP Extension, then the coupling ratio for this condition can be used as a baseline to assess the contribution of the windlass mechanism to the coupling ratio during other tasks. For example, the DFCR for Neutral heel raise was 6 times that of the seated passive condition. Thus, roughly 5/6 of that motion is likely attributable to influences other than the windlass mechanism (e.g. muscle contractions or energy storage and return).

Comparing seated and standing conditions along with active and passive MTP extension provided some insights into the influence of the windlass mechanism. Seated versus Standing had an effect on the coupling ratio but passive versus active did not. For active conditions, we expected the toe extensor muscles to exert a small dorsiflexion moment on the midtarsal joint, thus slightly reducing arch rise. This did not occur, and it appears that active recruitment of the dorsal foot muscles to extend the MTP joint (i.e. extensor hallucis longus and brevis) has little effect on arch rise, either because the moment arms of these muscles are too small or because co-contraction of plantar muscles negated their effect. In contrast, we noted a difference between seated and standing conditions, with the standing DFCR for both passive and active conditions smaller than both the seated DFCRs. Sichting and Ebrecht did not find a difference in the change in navicular height per degree of MTP extension between their seated and standing passive MTP extension conditions [23]. Possible explanations for these differences include measurement methodology (i.e. navicular height versus midtarsal angle as a measure of arch height) and potential differences in subject posture. We expect that the reason our standing conditions had smaller DFCR compared to seated may be due to the static loading experienced by the MLA during standing, which flattens the arch (Butler et al., 2008), increasing the distance between the MTP joint and calcaneus. This would either increase the tension in the PA or lengthen it, which could have an inhibiting or enhancing effect on the windlass mechanism [34]. The resting length of the PA was not measured, but if it were slack when seated, then weight bearing should have removed this slack and allowed MTP extension to raise the arch more effectively. However, since the DFCR dropped slightly when standing, this is likely not the case. If the PA is already tensioned while seated, weight bearing may increase that tension, but its effect on the windlass mechanism may depend on its extensibility. If it acts as a rigid cable, the additional tension from weight bearing should still raise the arch to the same extent per degree of MTP extension; however, if it is flexible, MTP extension may stretch the aponeurosis instead of raising the arch. Given that the DFCR was smaller during standing, the PA likely stretched in response to weight bearing rather than acting as a rigid cable. Additionally, a slight increase in the frictional resistance experienced by the calcaneus during standing compared to seated may have hindered the ability of the plantar aponeurosis to pull the calcaneus toward the metatarsal heads. Furthermore, the plantar intrinsic foot muscles provide postural support for the feet during standing [33], which may have increased midtarsal stiffness.

Arch rise was much greater during dynamic heel raises compared to the isolated MTP extension conditions, closely matching the results of Sichting and Ebrecht, who also found that arch rise was significantly greater during walking compared to passive MTP extension during sitting and standing [23]. The difference between dynamic and isolated MTP extension likely has a large contribution from the plantar intrinsic and extrinsic foot muscles. These muscles are active during the propulsive phase of walking [35, 36] and a likely source of tension across the arch [24]. As foot and ankle mechanics are similar between push-off of walking and heel raises [31], these muscles are likely active in a similar manner during heel raises. While we did not see any differences among the three heel raise conditions, this may be due in part to the statistical treatment, where we had numerous pairwise comparisons. Without an adjustment for multiple comparisons, ToeExt had a significantly larger DFCR than Neutral (*p* = 0.03), which became insignificant after adjustment (*p* = 0.13). With a larger sample size or more direct comparison between heel raise conditions, this relationship may have been significant. If weight bearing results in some stretch of the PA, starting heel raises with the toes extended should increase this stretch, potentially removing any extensibility and resulting in greater arch rise. As mentioned, when compared to the passive seated and passive standing conditions, only about 1/6 of the arch rise seen during heel raises is likely attributable to the windlass mechanism. Combined with the minimal effect of starting with the toes extended, it is likely that the windlass mechanism plays a secondary role in dynamic arch rise compared to the role of active muscle contractions.

### Kinetic coupling during heel raises

Although the kinematic coupling ratio was not statistically significant among the dynamic heel raise conditions, the amount of joint work at the MTP and midtarsal joints was substantially affected by the starting position of the MTP joint in these tasks. Starting with the toes extended resulted in a greater amount of work being absorbed at the MTP joint as well as generated at the midtarsal joint. Conversely, starting with the toes flexed resulted in less work being absorbed at the MTP joint and generated at the midtarsal joint. These results are in support of our hypothesis that the work generated at the midtarsal joint would change proportionally to the work absorbed at the MTP joint during heel raises (as indicated by the consistency of distal to hindfoot work across conditions). This hypothesis was based on previous investigations that found kinetic coupling in walking and running. As walking speed increases, both MTP negative work and midtarsal positive work increase [18]. Likewise, when comparing the power profiles of runners with varying foot strikes, Bruening et al. found that forefoot strikers had greater MTP negative work concurrent with greater midtarsal positive work [5].

Considering the results of the current study in conjunction with the work from which we formed our hypothesis, it is evident that there is a functional coupling between the MTP and midtarsal joints that may be even greater in kinetics than in kinematics. Yet, our results suggest that this kinetic coupling is likely due only in small measure to the windlass mechanism. Previous research has noted that during the propulsive phase of walking, the arch quickly rises despite a likely decrease in tension within the PA [34, 37], concurrent with continued MTP extension [38]. Biarticular plantar intrinsic and extrinsic foot muscles may play a large role, both actively and passively through spring-like properties. An investigation of the flexor digitorum brevis muscle (a biarticular muscle spanning the plantar aspect of the foot in parallel to the PA) found that during loading of the arch the muscle tendon stretches while the muscle fascicle is active isometrically [25]. During heel raises, the intrinsic foot muscles may be a source of power generation at the midtarsal joint and furthermore, stretch of these muscle tendons may facilitate energy storage and return between the MTP and midtarsal joints.

**Fig. 4:**
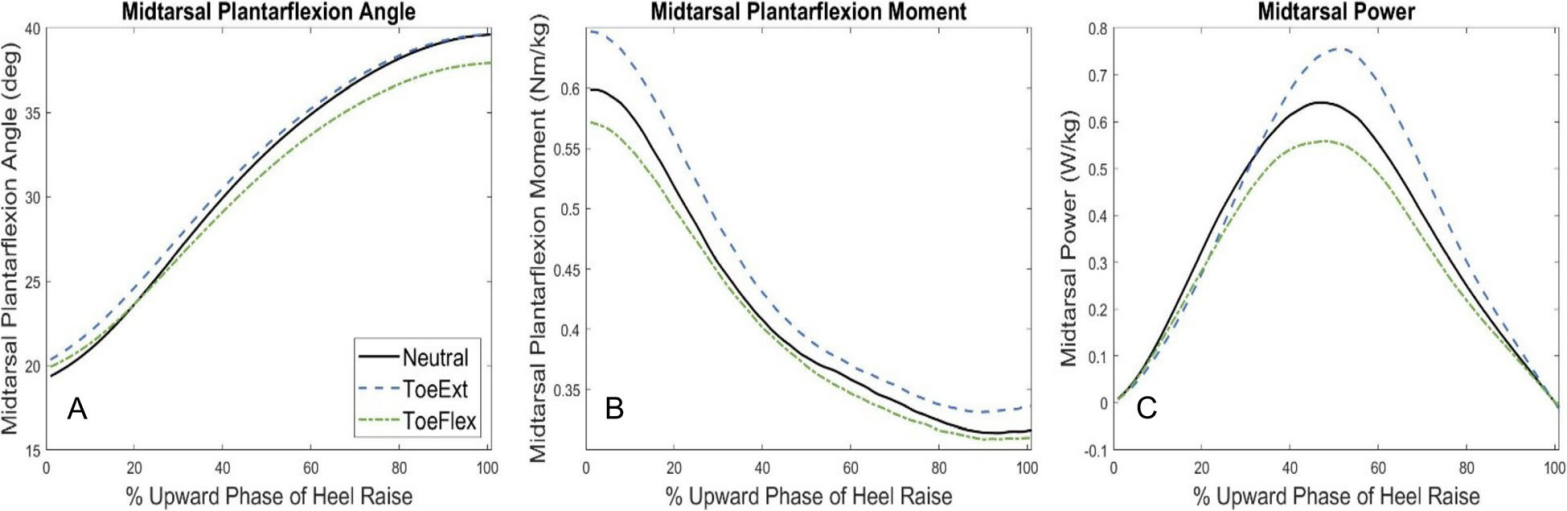
Midtarsal Angle, Moment, and Power During Heel Raise Conditions. Mean Midtarsal angle, moment, and power during the upward phase of Neutral (black, solid line), ToeExt (blue, dashed line), and ToeFlex (green, dash-dot line). A. Midtarsal plantar flexion angle; B. Midtarsal plantarflexion moment; C. Midtarsal power

The discrepancy between kinematics and kinetics effects in our study also suggests a large muscular role in distal foot coupling. To better understand the factors contributing to this coupling, we plotted the mean angle, moment, and power vs time graphs (Fig. 4) for each condition. For angles, although there was greater midtarsal plantarflexion in ToeExt at the start of heel raises, this did not result in a greater peak angle or angular velocity compared to Neutral (Fig. 4A). Instead, the midtarsal plantarflexion moment increased throughout the movement for ToeExt (Fig. 4B). This increased moment appears to be the main contributor to the increased peak power and work values in ToeExt (Fig. 4C) and is likely due to an anteriorly shifted center of pressure (COP). Shifting the COP in either an anterior or posterior direction should affect both MTP and midtarsal joints fairly equally, with associated proportional increases in active or passive tension at both joints and may be a main factor in this kinetic coupling. In addition, the altered joint moments could also change the muscle force-length positioning, with a more advantageous length in the ToeExt starting position. The ToeFlex condition, in contrast, exhibited less peak midtarsal plantarflexion and slightly lower moment compared to Neutral, perhaps due to being placed in a disadvantageous position. However, more research investigating the extent of energy transfer between these two joints could provide greater insight as it is still unclear if any of the energy absorbed at the MTP joint is transferred to the midtarsal joint through muscle tendons and the PA or simply dissipated.

### Arch flexibility and arch height versus kinematic coupling

We based our hypothesis that high arch flexibility and low arches would be related to a less efficient windlass mechanism (i.e., smaller DFCR) on the results from a study done by Lucas and colleagues [27]. In partial support of our hypothesis, there was a negative correlation between arch flexibility and DFCR during Seated Active MTP extension, with DFCR decreasing with increasing flexibility. Similarly, there was a negative correlation between arch height and DFCR during Standing Active MTP extension, with DFCR decreasing with increasing arch height. Contrary to our hypothesis, though, there was a positive correlation between arch flexibility and DFCR during both Neutral and ToeFlex heel raises. It is possible that the relationship between arch structure and the windlass mechanism is more prominent in isolated movements; however, the lack of significant correlations across all other isolated tasks suggests something more equivocal. The research exploring the relationship between static structure and dynamic foot function in walking and running is mixed. A number of researchers have found no correlation between foot structure and range of motion during stance (e.g. [39, 40]). Yet, isolated studies such as Magalhães et al. provide some reason for continued research. They found that individuals with greater foot mobility had increased range of motion in both the frontal and sagittal planes at the midfoot joint complex during walking compared to individuals with less foot mobility [41]. Thus, the relationship between foot structure and function is nuanced and warrants further investigation. Our results suggest that arch height or arch flexibility alone may not be adequate predictors of dynamic foot function.

A traditional clinical assumption is that high arches are stiff and low arches are flexible [42, 43]. However, recent work demonstrates that many arch flexibility types exist within arch height types [30]. The current study supports this notion, as we found that 21% of our participants had both stiff and low arches while 10% had both flexible and high arches. To classify the arches of our participants for this tally, we used the classifications of Zifchock et al. [30] for arch flexibility, grouping the ‘very-stiff’ and ‘stiff’ categories into one category called ‘stiff’ (similar grouping was done for the ‘very-flexible’ and ‘flexible’ categories). For arch height, the average of the cut-offs specified by Hillstrom et al. [44] and Williams et al. [45] was used. Perhaps if we recruited individuals that had both stiff and high arches or flexible and low arches, or more clinically extreme foot structures, a stronger correlation between foot structure and DFCR would have been observed. Future studies could explore these specific populations as it may provide useful insight for clinical applications.

There are some limitations to our work. First, our measure of arch flexibility calculated using the AHIMS arch height index measurement system may not be an accurate measure of functional arch flexibility as it is calculated from static positions. Future work could explore the relationship between foot function and arch flexibility or stiffness calculated during dynamic movement. Secondly, we did not control for possible anterior-posterior COP differences during our heel raise conditions. We had subjects focus on reaching the same height between conditions, which likely helped with any possible anterior-posterior leaning. However, it is possible that the COP was different between conditions which would affect inverse dynamic calculations. It may be possible to control the COP using real-time feedback, but this might also affect the heel raise motion. Future investigations could explore this possibility. Lastly, bilateral heel raises may not correlate to walking quite as well as single leg heel raises [31]. However, we felt that bilateral heel raises were needed in this study to reduce balance hurdles and better control movements between the different heel raise conditions.

## Conclusions and applications

When MTP motion is systematically manipulated during heel raises, the changes in midtarsal positive work and MTP negative work change proportionally. This indicates that there is kinetic coupling between these two joints. Furthermore, the amount of kinematic coupling within the distal foot increased substantially during heel raises compared to when the MTP joint was passively extended in a non-weight-bearing position. Thus, if the windlass mechanism influences power generation at the midtarsal joint, it is likely a small role secondary to active muscle contractions or other mechanisms. Foot coupling could be partially due to energetic transfer between the distal and midfoot, facilitated by biarticular muscles and ligaments [5, 19]. However, the extent of this energy transfer is still unclear and requires further investigation. Particularly, there is a need for further research involving measures of muscle contractions within the foot during dynamic tasks as well as research into the resting length and force-length properties of the intrinsic foot muscles and the resting length and extensibility of the PA. Lastly, the relationship between foot structure and function is still unclear and our results suggest that arch height or arch flexibility alone may not be adequate predictors of dynamic foot function.

This work sets the foundation for informing the design of assistive foot devices. The plantar flexor muscles at the ankle have historically been attributed as the primary generators of the power used for propulsion [46–49], and design of assistive foot devices often aims to reproduce the energy generated at the ankle. But, multisegment foot studies have shown that this ankle power has been overestimated and between 27 and 66% [11, 50, 51] of it should be attributed to the midtarsal joint instead, indicating that the midtarsal joint actively contributes to push-off. If some of the energy absorbed at the MTP joint is transferred to the midtarsal joint, implementation of this energetic transfer into the design of assistive devices could be advantageous. For instance, adding stiff insoles into running shoes has improved both distance running and sprinting performance [52, 53], although the exact mechanisms behind this augmentation are not fully understood. Two likely possibilities include increased energy storage/return and an anteriorly shifted center of pressure which would alter muscle actions within the foot [54]. Carrier et al. [55] referred to the latter as an altered “gearing ratio,” i.e. the external ground reaction force moment arm relative to the plantar flexor muscle moment arm about the ankle. Our results suggest that this gearing concept should be expanded to the joints and muscles of the mid and forefoot. In prosthetics, implementing a toe joint into the design of prosthetic feet has shown that altering foot properties affects center of mass push-off dynamics, perhaps even more than when ankle properties are adjusted [56, 57]. Additional insight into the mechanisms behind these findings could further enhance orthoses for running or clinical populations with propulsion deficits. For the latter, there is currently a knowledge gap in the design of foot orthoses that enhance walking function for patients, and the results of this study (namely the kinetic coupling) support the potential of novel deformable or energy-storing-and-returning foot orthoses to enhance foot energetics during walking. Future research should investigate whether external devices such as orthoses can be integrated into the distal foot joint coupling seen in the natural foot or whether it will be offset by compensations elsewhere in the ankle/foot system.

## Supplementary Information

**Additional file 1.** Marker set description and model details This additional file contains details about the multisegment foot marker set used for this study. It includes marker description of placement, images showing the marker set from 4 different views, and details about model creation.

## Data Availability

The datasets used and/or analyzed during the current study are available from the corresponding author on reasonable request.

## Abbreviations

MTP: Metatarsophalangeal
PA: Plantar aponeurosis
AH: Arch height, height of the foot’s dorsum from the floor at half of the total foot length
BW: Body weight
DFCR: Distal foot coupling ratio, ΔMidtarsal Angle / ΔMTP Angle
ANOVA: Analysis of variance
