# Supplementary material for: The influence of the windlass mechanism on kinematic and kinetic foot joint coupling
Source: J Foot Ankle Res. 2022 Feb 16;15:16. doi: 10.1186/s13047-022-00520-z (PMC8848977; doi:10.1186/s13047-022-00520-z)
Supplement: Supplementary file 1 — Additional file 1. Marker set description and model details This additional file contains details about the multisegment foot marker set used for this study. It includes marker description of placement, images showing the marker set from 4 different views, and details about model creation. [file 13047_2022_520_MOESM1_ESM.pdf]

Table S1. Marker Names and Description of Placement

Marker Name	Description of Placement
HEAD	Top of the head
LKN	Lateral epicondyle of femur
MKN	Medial epicondyle of femur
SHK1-4*	Shank cluster 1-4; rigid cluster on lateral shank
LANK	Lateral malleolus
MANK	Medial malleolus
PCL*	Proximal calcaneus
DCL*	Distal calcaneus; apex of calcaneal tuberosity
LCL*	Lateral calcaneus
MCL*	Medial calcaneus
CUB	Cuboid; lateral cuboid
NAV	Navicular; navicular tuberosity
MB1	Dorsal surface of the base of the 1st metatarsal
MB4	Dorsal surface of the base of the 4th metatarsal
MHD	Dorsal surface 1st metatarsal head; dorsum of head of the 1st metatarsal
MH1	1st Metatarsal head; medial aspect of head of the 1st metatarsal
23MH	2nd and 3rd Metatarsal heads; midpoint of heads of the 2nd and 3rd metatarsals
MH5	5th Metatarsal head; lateral aspect of head of the 5th metatarsal
HAL	Hallux; dorsum of the distal phalanx of the hallux

*Tracking Marker

Figure S1. Dorsal, lateral, medial, and posterior views of multisegment foot markers. Marker names are abbreviated. Full marker name and description of placement can be found in Table S1.

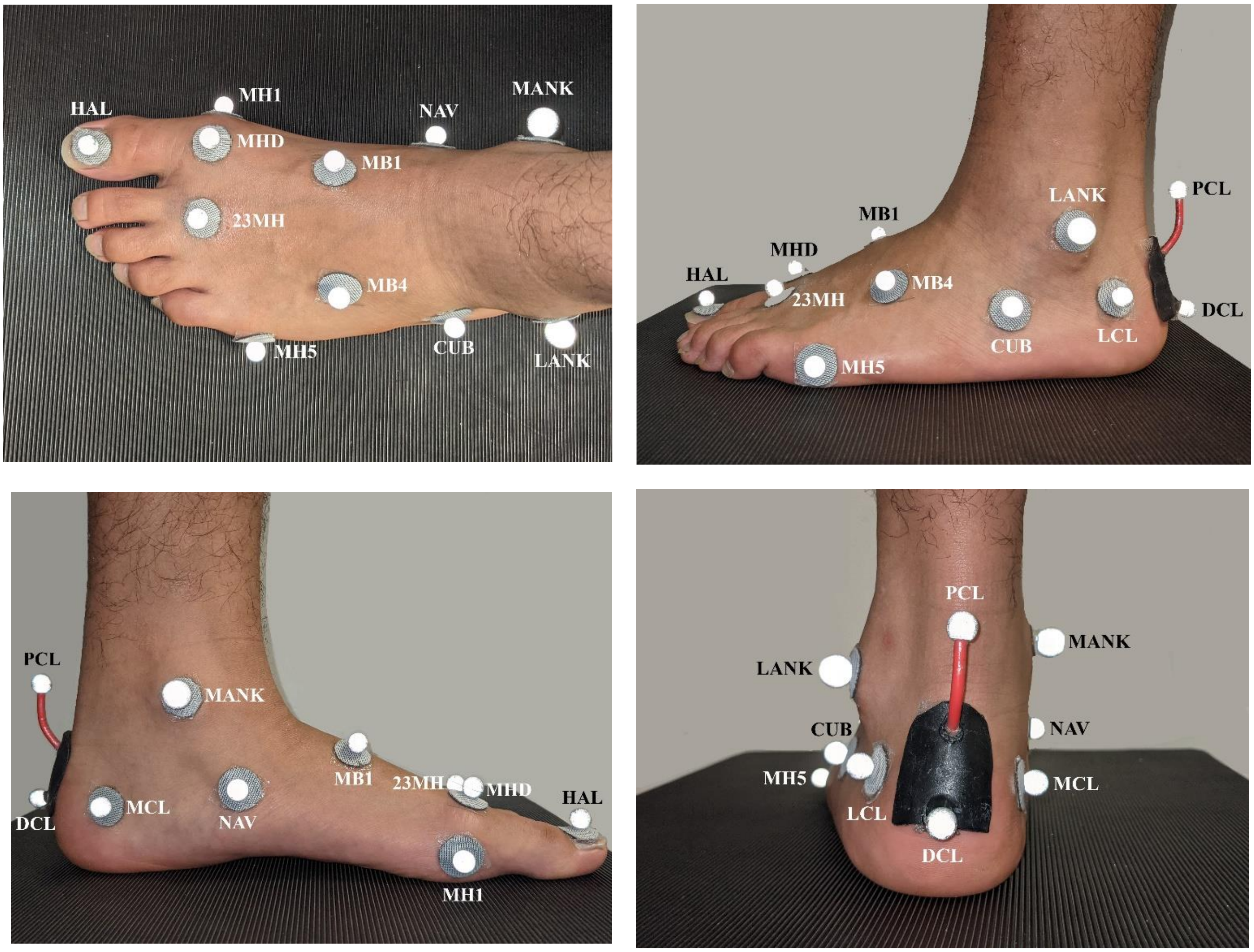

Table S2. Virtual Landmarks for Multi-segment Foot Model

MJC	Midtarsal joint center – midpoint between CUB, NAV
FF_Dist	Forefoot distal end – project 23MH down to average height of MH1 and MH5
MTPC	1st MTP joint center – project MHD ½ distance to floor
HAL_Dist	Hallux distal end – project HAL ½ distance to floor
MTPC_Track	MTPC tracked by forefoot segment
MHD_Track	MHD tracked by forefoot segment
MHD_Proj	MHD_Track projected laterally in plane of HAL, MTPC_Track, MHD_Track

Table S3. Multi-segment foot model segment reference frame definitions

Segment	Long Axis	Plane	Tracking Markers
Hindfoot	DCL to MJC	DCL, MJC, PCL (Sagittal)	DCL, MCL, LCL
Forefoot	MJC to FF_Dist	MJC, FF_Dist, 23MH (Sagittal)	MB1, MB4, 23MH, MH1, MH5
Hallux	MPC to HX_Dist	MPC, HAL_Dist, MH1 (Sagittal)	HAL, MTPC_Track, MHD_Proj
